# Comparative Proteomics Analysis Reveals L-Arginine Activates Ethanol Degradation Pathways in HepG2 Cells

**DOI:** 10.1038/srep23340

**Published:** 2016-03-17

**Authors:** Guokai Yan, Retno Lestari, Baisheng Long, Qiwen Fan, Zhichang Wang, Xiaozhen Guo, Jie Yu, Jun Hu, Xingya Yang, Changqing Chen, Lu Liu, Xiuzhi Li, Agung Purnomoadi, Joelal Achmadi, Xianghua Yan

**Affiliations:** 1 College of Animal Sciences and Technology, Huazhong Agricultural University, Wuhan, 430070, Hubei, China; 2 The Cooperative Innovation Center for Sustainable Pig Production, Wuhan, 430070, Hubei, China; 3 Faculty of Animal and Agricultural Sciences, Diponegoro University, Tembalang Campus, Semarang 50275, Central Java, Indonesia; 4 State Key Laboratory of Agricultural Microbiology, College of Veterinary Medicine, Huazhong Agricultural University, Wuhan, 430070, Hubei, China

## Abstract

L-Arginine (Arg) is a versatile amino acid that plays crucial roles in a wide range of physiological and pathological processes. In this study, to investigate the alteration induced by Arg supplementation in proteome scale, isobaric tags for relative and absolute quantification (iTRAQ) based proteomic approach was employed to comparatively characterize the differentially expressed proteins between Arg deprivation (Ctrl) and Arg supplementation (+Arg) treated human liver hepatocellular carcinoma (HepG2) cells. A total of 21 proteins were identified as differentially expressed proteins and these 21 proteins were all up-regulated by Arg supplementation. Six amino acid metabolism-related proteins, mostly metabolic enzymes, showed differential expressions. Intriguingly, Ingenuity Pathway Analysis (IPA) based pathway analysis suggested that the three ethanol degradation pathways were significantly altered between Ctrl and +Arg. Western blotting and enzymatic activity assays validated that the key enzymes ADH1C, ALDH1A1, and ALDH2, which are mainly involved in ethanol degradation pathways, were highly differentially expressed, and activated between Ctrl and +Arg in HepG2 cells. Furthermore, 10 mM Arg significantly attenuated the cytotoxicity induced by 100 mM ethanol treatment (*P* < 0.0001). This study is the first time to reveal that Arg activates ethanol degradation pathways in HepG2 cells.

L-Arginine (Arg), a conditionally essential amino acid for adult mammals, serves as one of the building blocks for proteins and peptides synthesis, and plays crucial roles in multiple physiological processes including spermatogenesis, embryonic survival, fetal and neonatal growth, maintenance of vascular tone and hemodynamics^1^. In mammals, Arg is also responsible for the production of nitric oxide (NO), creatine, and polyamines (putrescine, spermidine, and spermine)^2^, which have been proved to act as vital regulators for the synthesis of DNA and proteins^3^, scavenging of reactive oxygen species (ROS)^4^, inhibition of autophagy^5^, cell proliferation^6^,^7^,^8^, and fat metabolism^9^,^10^. Moreover, several lines of studies suggest that dietary Arg supplementation could alter various performances of animals. For instance, Tan *et al*. found that dietary Arg supplementation enhances the immunity of early-weaned piglets^11^. Meanwhile, increased muscle gain and decreased body fat mass of growing-finishing pigs were observed^12^. Ren *et al*.^13^ also found that dietary Arg supplementation could lead to the activation of intestinal innate immunity in mice^13^.

To gain a comprehensive understanding of the effect of amino acids deprivation and/or supplementation, 2-dimensional gel electrophoresis (2-DE) based proteomics approaches have been commonly applied in functional studies of amino acids. For instance, Lenaerts *et al*.^14^ analyzed the effect of glutamine on protein profile of Caco-2 cells using a strategy combining 2-DE and MALDI-TOF-MS and identified 14 differentially expressed proteins specially altered by glutamine^14^. Xin and co-workers investigated the molecular mechanisms of cell cycle arrest and apoptosis in response to methionine restriction by comparing the proteomic alterations between gastric cancer cells SGC7901 cultured in methionine-complete medium or methionine-depleted homocysteine-supplemented medium using an approach combining 2-DE and MALDI-TOF-MS^15^. Using the same approach, Lenaerts *et al*.^16^ explored the protein profiles induced by Arg deficiency in either preconfluent or postconfluent Caco-2 cells and found that Arg deprivation leads to the decreased cell proliferation and heat shock protein expression, and the enhanced cell susceptibility to apoptosis^16^.

Developed by Applied Biosystems Incorporation (ABI) in 2004, isobaric tags for relative and absolute quantification (iTRAQ), which shows many advantages with respect to high-throughput, high sensitivity, and great accuracy, has grown to be a robust technique in comparative proteomics field^17^. To our knowledge, there are no reports focusing on the proteome scale alteration caused by Arg in liver cells. Here, in this study, iTRAQ-based proteomic approach was conducted to characterize the proteome profile between the Arg-deprived (Ctrl) and Arg-supplemented (+Arg) HepG2 cells (Fig. 1A). Bioinformatics analysis revealed that several differentially expressed proteins were involved in crucial biological processes including amino acid metabolism. Most importantly, our data indicated that ethanol degradation pathways were significantly activated in Arg-supplemented HepG2 cells, suggesting the potential role of Arg on ethanol degradation in HepG2 cells.

## Results

### Quantitative proteome analysis of HepG2 cells treated with Arg supplementation

**Figure 1:**
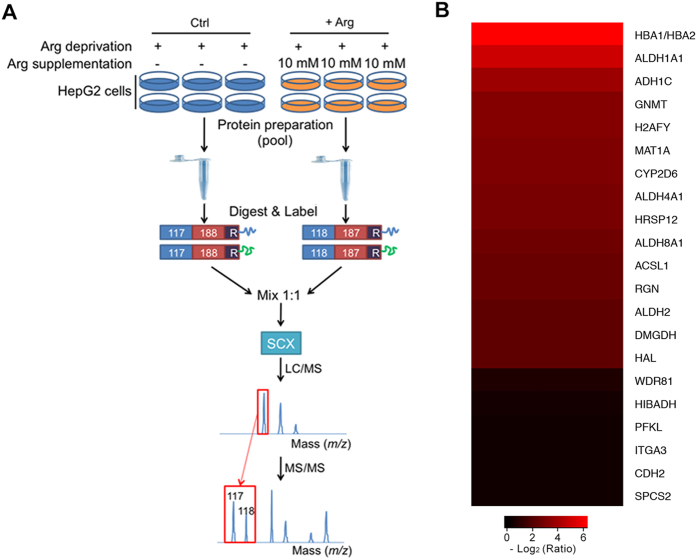
Schematic diagram of workflow for the iTRAQ-based experiments. Six replicates were pooled into one sample for either Arg-deprived (Ctrl) or Arg-supplemented (10 mM) (+Arg) HepG2 cells. Then pooled cells were fractionated, digested into peptides with trypsin, and the peptides are labeled with different iTRAQ reagents, which contain reporter groups of different masses (117, 118), balance groups of different masses (188, 187), and a reactive group (R). The labeled peptides are then mixed equivalently, and fractionated by strong cation exchange (SCX) chromatography. Fractions were separated by liquid chromatography (LC) and analyzed by two-step mass spectrometry (MS). Two independent biological replicates were performed to increase the statistical confidence. (**B**) Differentially expressed proteins identified by iTRAQ analysis of HepG2 cells treated by Arg supplementation. More information is available in Supplementary Table S1.

By LC-MS/MS analysis, a total of 4,209 proteins were quantified between Ctrl and +Arg in the two sets of biological replicates (Supplementary Fig. S1). Further analysis identified 21 proteins as differentially expressed proteins and they were all up-regulated by Arg supplementation (Fig. 1B and Supplementary Table S1).

**Figure 2:**
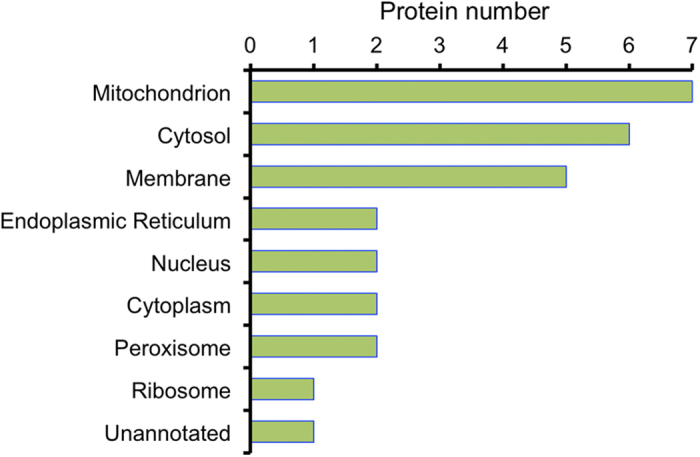
Gene Ontology (GO) subcellular annotation of differentially expressed proteins in Arg-supplemented HepG2 cells. Horizontal axis means the actual number of differentially expressed protein annotated in certain compartment.

We next aimed to catalogue the subcellular annotation of the regulated proteins. Based on the Gene Ontology (GO), molecular classification of cellular component was shown in Fig. 2, the differentially expressed proteins were found to mainly localize to the mitochondrion, cytosol, and membrane.

**Figure 3:**
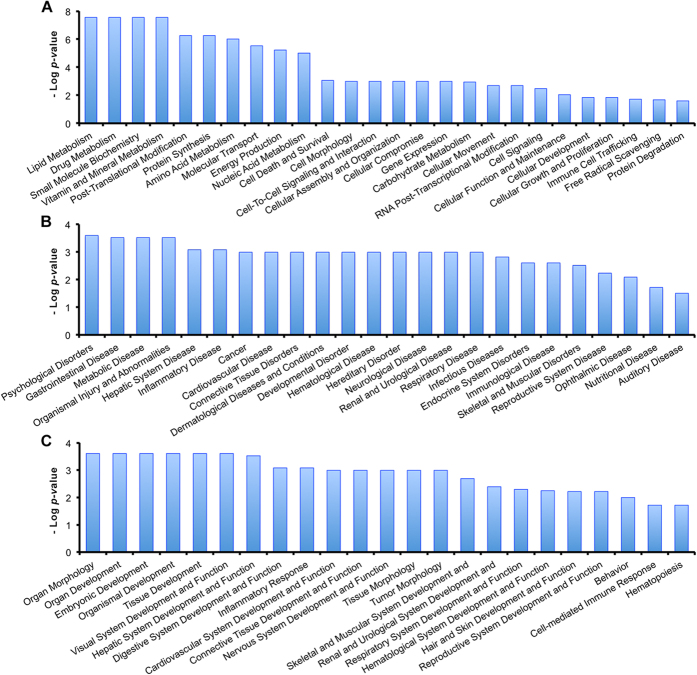
Functional characterization of all the differentially expressed proteins. (**A**) Molecular and cellular functions. (**B**) Diseases and disorders. (**C**) Physiological system development and functions. More information is available in Supplementary Table S2.

To better analyze the function characterization and biological processes of relevant proteins, the Uniprot accession numbers and ratios (+Arg/Ctrl) of all differentially expressed proteins were uploaded into the Ingenuity Pathway Analysis (IPA) software. According to the functional enrichment analysis of IPA, all the uploaded proteins could be classified into three diverse functional settings: molecular and cellular functions, diseases and disorders, and physiological system development and functions. As shown in Fig. 3 and Supplementary Table S2, differentially regulated proteins were all characterized with statistical significance (*P* < 0.05). The differentially expressed proteins were shown to be involved in various biological processes, such as “Lipid Metabolism” and “Amino Acid Metabolism” (Fig. 3A), “Hepatic System Disease” (Fig. 3B), and “Organ Morphology” (Fig. 3C).

**Figure 4:**
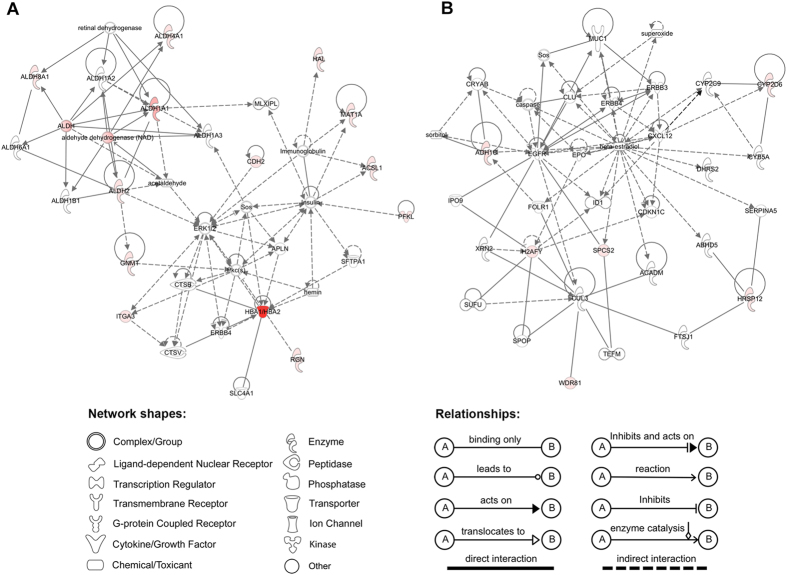
Network analysis of differentially expressed proteins altered in Arg-supplemented HepG2 cells using the IPA tools. (**A**) Drug metabolism, energy production, lipid metabolism. (**B**) Tissue development, cellular development, cell growth and proliferation. Red, significantly up-regulated proteins; green, significantly down-regulated proteins; white, proteins are involved in certain network but not differentially expressed in this study. The degree of change for protein expression is indicated by color depth.

A large number of proteins can only be brought into effect through interacting with other partners, studying the interacting relationship among all the differentially expressed proteins is meaningful to understand the integral biological role of relevant proteins. Thus, we also used the IPA tool to perform the network analysis that based on the microarray results of published literatures^18^. All the differentially expressed proteins were shown to be involved in two networks. Based on the scores, the two networks are: 1) drug metabolism, energy production, lipid metabolism (score, 33; Fig. 4A); 2) tissue development, cellular development, cell growth and proliferation (score, 13; Fig. 4B).

### Arg supplementation induces the alteration of amino acid metabolism-related proteins in HepG2 cells

It is noteworthy that in the function characterization of IPA analysis, amino acid metabolism was significantly altered (*P* value 9.69e^−7^; Fig. 3A). As shown in Table 1, a total of six differentially expressed proteins, such as S-adenosylmethionine synthase (MAT1A, 8.566-fold) and 3-hydroxyisobutyrate dehydrogenase (HIBADH, 1.428-fold), were involved in the metabolisms of various amino acids including cysteine and methionine metabolism, branched amino acid (valine, leucine, and isoleucine) degradation.

### Ethanol degradation pathways are significantly activated by Arg supplementation

**Figure 5:**
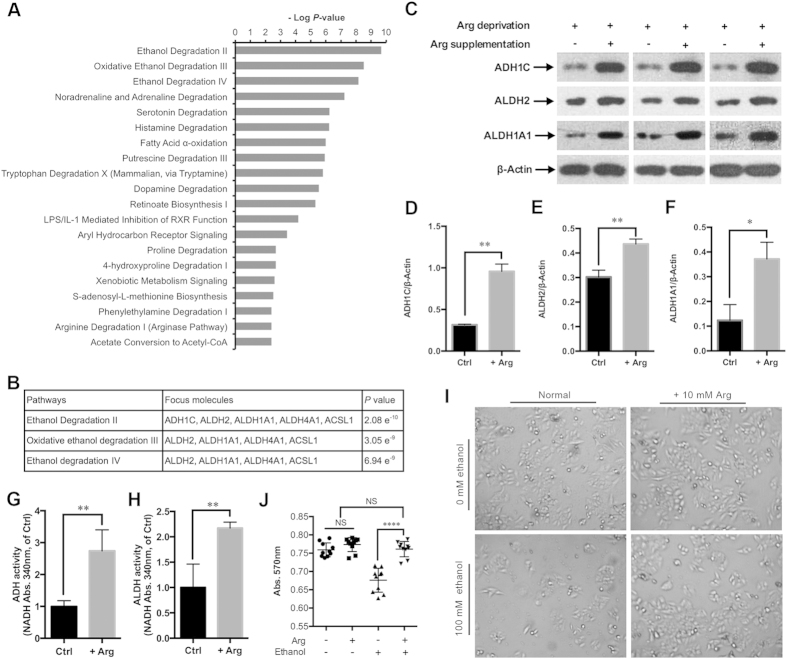
Arg supplementation activates ethanol degradation pathways in HepG2 cells. (**A**) The 20 top-ranked canonical pathways based on *P*-values by the IPA tools. (**B**) Detailed information of the three top-ranked canonical pathways. (**C**) Western blot of ADH1C, ALDH1A1, ALDH2, and β-Actin in the HepG2 cells treated by Arg deprivation and Arg supplementation (10 mM) as indicated. (**D–F**) Quantification of ADH1C/β-Actin, ALDH2/β-Actin, and ALDH1A1/β-Actin as described in (**C**), respectively. Data are means ± SD (n = 3). ^*^P < 0.05, ^**^P < 0.01 (Student’s *t*-test). (**G,H**) The ADH and ALDH activity in Arg-deprived (Ctrl) and Arg-supplemented (+Arg) HepG2 cells (compared to that of Ctrl) were determined by measuring the rate of NADH production at 340 nm. Data are means ± SD (n = 4). ^**^P < 0.01 (Student’s *t*-test). (**I**) The morphological changes of the ethanol (100 mM) and/or Arg (10 mM)-treated (24 h) HepG2 cells were observed by light microscopy. (**J**) The cytotoxicity of ethanol and/or Arg-treated HepG2 cells (as described in I) was measured by MTT assay. Data are means ± SD (n = 10). NS, non-significant, ^****^P < 0.0001 (Student’s *t*-test).

To get biological insight into the alteration of signaling pathways induced by Arg supplementation, the IPA tool was also used to study the canonical pathways all the differentially expressed proteins involved. Some crucial signaling pathways were altered by Arg supplementation, such as the “Fatty Acid α-Oxidation” (*P* = 1.03e^−6^), and the “Signaling by Rho Family GTPases” (*P* = 2.34e^−2^). In addition, among all the canonical pathways ranked by *P*-values, the three ethanol degradation pathways (Ethanol Degradation II, Oxidative Ethanol Degradation III, and Ethanol Degradation IV) were shown to harbor minimum *P*-values (Fig. 5A). Three proteins alcohol dehydrogenase 1C (ADH1C, 14.530-fold), retinal dehydrogenase 1 (ALDH1A1, 32.124-fold), and aldehyde dehydrogenase 2 (ALDH2, 5.305-fold), which are the key enzymes involved in the ethanol degradation pathways, were up-regulated by Arg supplementation (Fig. 5B). To validate the LC-MS/MS data and confirm whether ethanol degradation pathways are altered by Arg supplementation, we compared the ratios of ADH1C, ALDH1A1, and ALDH2 between Ctrl and +Arg in HepG2 cells by western blotting (Fig. 5C). Statistics analysis (*t*-test) of the data confirmed that the ratios of these three proteins were consistent with those determined using the iTRAQ labeled LC-MS/MS strategy (Fig. 5D–F). Meanwhile, the ADH1C, ALDH1A1, and ALDH2 were down-regulated in Arg depletion condition compared to normal medium-cultured HepG2 cells (Supplementary Fig. S2). Furthermore, the enzymatic activities of ADH, ALDH were increased by Arg supplementation (Fig. 5G,H). 10 mM Arg supplementation significantly attenuated the cytotoxicity induced by 100 mM ethanol in HepG2 cells (Fig. 5I,J). These results suggest that Arg supplementation leads to the activation of the ethanol degradation pathways in HepG2 cells.

## Discussion

In an effort to identify the differentially expressed proteins related to Arg supplementation in HepG2 cells, we employed the iTRAQ-based comparative proteomic approach. One of our major findings was that Arg supplementation can activate the ethanol degradation pathways in HepG2 cells. As liver serves as a main organ in ethanol metabolism, ethanol is largely metabolized to acetaldehyde by alcohol dehydrogenases (ADH) in the cytosol of liver cells^19^. Then the acetaldehyde, which can cause highly toxic effect in organism functions or induce a series of behavioral effects^20^,^21^, is converted by aldehyde dehydrogenases (ALDH) to acetate^19^,^22^. Previously, a number of studies have demonstrated that ethanol oxidation can be accelerated by specific amino acids. For instance, alanine was found to have ability to accelerate ethanol oxidation in acute ethanol treated rats livers^23^; leucine was found to enhance the activity of several ethanol metabolic enzymes including ADH and low Km ALDH in the livers of spontaneously hypertensive stroke prone (SHRSP) rats, thus leading to the acceleration of blood ethanol oxidation^24^. However, to our knowledge, there is no literature reported that Arg functions in ethanol oxidation. It is striking that in the data derived from iTRAQ experiment, ALDH1A1 and ALDH2, two main ALDHs that degrade acetaldehyde in human liver^25^,^26^, showed significant up-regulations by fold-changes of 32.124 and 5.305 after 30 min of Arg supplementation, respectively (Supplementary Table S1). Meanwhile, the network analysis revealed that ALDH1A1 and ALDH2 were involved in the most enriched network “drug metabolism, energy production, lipid metabolism”, and can directly interact with each other (Fig. 4A), suggesting that these two enzymes might be effective through interacting with each other upon Arg supplementation. Moreover, the alcohol catalase ADH1C (previously known as ADH3), which is response to the alcohol catabolism in liver^26^,^27^,^28^, showed a fold-change of 14.530 (Supplementary Table S1). Furthermore, the immunoblotting and enzymatic activity assays validated the differential expressions, and showed the increasing enzymatic activities of these three key enzymes in Arg-supplemented HepG2 cells, respectively. Importantly, Arg significantly reduced the cytotoxicity induced by 100 mM ethanol in HepG2 cells (*P* < 0.0001). These findings suggest that Arg may play crucial roles in ethanol metabolism in HepG2 cells. It is still unknown why Arg supplementation leads to the up-regulation of ADH1C, ALDH1A1, and ALDH2. However, it has been shown that a part of endogenous ethanol can be produced by amino acid metabolism. Thus it is possible that Arg stimulates the up-regulation of ethanol degradation-related enzymes by directly or indirectly producing endogenous ethanol in HepG2 cells.

It is unequivocal that amino acid metabolism can be affected by each other, for instance, metabolism of some amino acids can contribute to the *de novo* synthesis of non-essential amino acids^29^. In this study, the bioinformatics analysis showed the effect of Arg supplementation on the metabolism of other amino acids. We identified six differentially expressed proteins that are involved in both catabolism and anabolism of amino acids (Table 1). Among these six differentially expressed proteins, MAT1A is an enzyme only exists in human hepatocytes and is responsible for the synthesis of S-adenosylmethionine, the main methyl donor and a precursor in the synthesis of key metabolites such as glutathione and polyamines^30^,^31^,^32^. Intriguingly, in this study, MAT1A was up-regulated by Arg supplementation, suggesting a potential way for Arg regulating methionine and methyl metabolisms in HepG2 cells.

Besides, our iTRAQ data have shown several crucial proteins were significantly altered by Arg supplementation. The liver-type 6-phosphofructokinase (PFKL), a master regulator of glycolysis^33^, was up-regulated by 1.396-fold, which suggested that the glycolysis may be up-regulated by Arg supplementation. Intriguingly, several previous studies have indicated that ethanol can inhibit hepatic gluconeogenesis^34^,^35^,^36^. Our results, however, suggested that the metabolism of hepatic glucose was affected by another pathway (glycolysis) that might be induced by ethanol metabolism after Arg supplementation. RHOA signaling plays multiple roles in regulating cell functions, such as regulating the formation of F-actin stress fibers and focal adhesion complexes^37^,^38^. Several studies have suggested that the RHOA pathway can be regulated by NO^39^,^40^. In addition, Krepinsky and colleagues found that dietary Arg supplementation can lead to the RHOA inactivation by increasing the amount of NO in rat mesangial cells^41^. However, in our study, RHOA was likely up-regulated (fold-change of 1.360 in one set of iTRAQ experiment) by Arg supplementation in HepG2 cells. Moreover, the proteins cadherin-2 (CDH2, 1.339-fold) and integrin alpha-3 (ITGA3, 1.341-fold), which have been proved to be involved in the pathway of signaling by Rho family GTPases^38^, were also showed up-regulations by Arg supplementation. We speculated that Arg might have different effects on the RHOA signaling in various cell types by different mechanisms, one of which is by producing NO.

In summary, we characterized the proteome alteration induced by Arg supplementation using a strategy combining iTRAQ with LC-MS/MS in HepG2 cells. Our study has shown that the metabolism of several amino acids may be altered by Arg supplementation and Arg supplementation can activate the three ethanol degradation pathways through enhancing the expressions and enzymatic activities of three key enzymes, ADH1C, ALDH1A1, and ALDH2. Our findings provide new insight into the role of Arg on ethanol degradation. However, further research still needs to elucidate that Arg can activate the ethanol pathways in various liver cell lines and animal livers. This will contribute to attenuate acute and/or chronic malfunction caused by ethanol in liver using functional amino acids, especially arginine.

## Materials and Methods

### Cell line and culture

The human liver hepatocellular carcinoma (HepG2) cell line was kindly provided by Dr. Zaiqing Yang (Huazhong Agricultural University, College of Life Science and Technology). The medium used in this study was RPMI-1640 without arginine, leucine, and lysine (R1780, Sigma Aldrich). To produce the Arg-deprived medium, 0.8 mM leucine (E811, Amresco) and 0.8 mM lysine (M234, Amresco) were added into the medium containing 10% fetal bovine serum (1660516, Gibco) and 1% penicillin-streptomycin (15070, Invitrogen). For Arg deprivation treatment, cells were cultured in Arg-deprived medium for 80 min at 37 °C under 5% CO_2_. For Arg supplementation treatment, 10 mM Arg (0953, Amresco) was added into the Arg-deprived medium for 30 min after 50 min Arg deprivation treatment.

### Protein preparation, protein digestion, and iTRAQ labeling

To reduce the sample variability, six replicates of harvested HepG2 cells were pooled into one sample for each treatment following the examples of previous studies^42^,^43^. Then the cells were suspended in the lysis buffer (7 M Urea, 2 M Thiourea, 4% CHAPS, 40 mM Tris-HCl, pH 8.5, 1 mM PMSF, 2 mM EDTA) and sonicated in ice. The proteins were reduced with 10 mM DTT at 56 °C for 1 h, alkylated by 55 mM IAM in the darkroom for 1 h. The protein mixtures were precipitated by adding 4× volume of chilled acetone at −20 °C overnight. After centrifugation at 4 °C, 30,000 g, the pellet was dissolved in 0.5 M TEAB (Applied Biosystems, Milan, Italy) and sonicated in ice. After centrifuging at 30,000 g at 4 °C, an aliquot of the supernatant was taken for determination of protein concentration by Bradford assay. Total protein (100 μg) was taken out of each sample solution and then digested with Trypsin Gold (Promega, Madison, WI, USA) with the ratio of protein : trypsin = 30:1 at 37 °C for 16 h. After trypsin digestion, peptides were dried by vacuum centrifugation. Peptides were reconstituted in 0.5 M TEAB and processed according to the manufacture’s protocol (Applied Biosystems). Briefly, one unit of iTRAQ reagent was thawed and reconstituted in 24 μL isopropanol. Samples were labeled with the iTRAQ tags as follow: Arg deprivation (Ctrl, tag 117), Arg supplementation (+Arg, tag 118), two independent biological replicates were performed to avoid the labeling bias. The peptides were then incubated at room temperature for 2 h. Followed by vacuum centrifugation.

### Strong cation exchange

Strong cation exchange (SCX) chromatography was performed with an LC-20AB HPLC Pump system (Shimadzu, Kyoto, Japan). The iTRAQ-labeled peptide mixtures were reconstituted with 4 mL buffer A (25 mM NaH_2_PO_4_ in 25% ACN, pH 2.7) and loaded onto a 4.6 × 250 mm Ultremex SCX column containing 5-μm particles (Phenomenex). The peptides were eluted at a flow rate of 1mL/min with a gradient of buffer A for 10 min, 5–60% buffer B (25 mM NaH_2_PO_4_, 1 M KCl in 25% ACN, pH 2.7) for 27 min, 60–100% buffer B for 1 min. The system was then maintained at 100% buffer B for 1 min before equilibrating with buffer A for 10 min prior to the next injection. Elution was monitored by measuring the absorbance at 214 nm, and fractions were collected every 1 min. The eluted peptides were pooled into 20 fractions, desalted with a Strata X C18 column (Phenomenex) and vacuum-dried.

### LC-ESI-MS/MS analysis based on Q EXACTIVE

Each fraction was resuspended in buffer A (2% ACN, 0.1% FA) and centrifuged at 20,000 g for 10 min, the final concentration of peptide was about 0.5 μg/μL on average. 10 μL supernatant was loaded on an LC-20AD nanoHPLC (Shimadzu, Kyoto, Japan) by the autosampler onto a 2 cm C18 trap column. Then the peptides were eluted onto a 10 cm analytical C18 column (inner diameter 75 μm) packed in-house. The samples were loaded at 8 μL/min for 4 min, then the gradient was run at 300 nL/min for 44 min at 2–35% B (98% ACN, 0.1% FA), followed by running at linear gradient to 80% for 2 min, and maintained at 80% B for 4 min, and finally returned to 5% in 1 min. The peptides were subjected to nanoelectrospray ionization followed by tandem mass spectrometry (MS/MS) in a Q EXACTIVE (Thermo Fisher Scientific, San Jose, CA) coupled online to the HPLC. Intact peptides were detected in the Orbitrap at a resolution of 70,000. Peptides were selected for MS/MS using high-energy collision dissociation (HCD) operating mode with a normalized collision energy setting of 27.0 (±12%); ion fragments were detected in the Orbitrap at a resolution of 17,500. A data-dependent procedure that alternated between one MS scan followed by 15 MS/MS scans was applied for the 15 most abundant precursor ions above a threshold ion count of 20,000 in the MS survey scan with a following Dynamic Exclusion duration of 15 s. The electrospray voltage applied was 1.6 kV. Automatic gain control (AGC) was used to optimize the spectra generated by the orbitrap. The AGC target for full MS was 3e^6^ and 1e^5^ for MS/MS. For MS scans, the *m/z* scan range was 350 to 2,000 Da. For MS/MS scans, the *m/z* scan range was 100–1,800.

### Bioinformatics analysis

Raw data files acquired from the mass spectrometers were converted into MGF files using 5600 msconverter, and the MGF files were searched. Protein identification was performed by using Mascot search engine (Matrix Science, London, UK; version 2.3.02) against Uniprot homo database (containing 143,397 sequences). For protein identification, a mass tolerance of 20 ppm was permitted for intact peptide masses and 0.05 Da for fragmented ions, with allowance for one missed cleavages in the trypsin digests. Gln → pyro-Glu (N-term Q), oxidation (M), deamidated (NQ) as the potential variable modifications, and carbamidomethyl (C), iTRAQ8plex (N-term), iTRAQ8plex (K) as fixed modifications. The charge states of peptides were set to +2 and +3. Specifically, an automatic decoy database search was performed in Mascot by choosing the decoy checkbox in which a random sequence of database is generated and tested for raw spectra as well as the real database. To reduce the probability of false peptide identification, only peptides with significance scores (≥20) at the 99% confidence interval by a Mascot probability analysis greater than “identity” were counted as identified. And each confident protein identification involves at least one unique peptides. The quantitative protein ratios were weighted and normalized by the median ratio in Mascot. To consider the differential expressions of proteins, we used the criteria as follow, 1) *P*-value < 0.05, fold change > 1.2 (or <0.833) in both the two sets of iTRAQ experiments and mean fold change > 1.3 (or <0.769); 2) *P*-value < 0.05, fold change > 5 (or <0.2) in one set of iTRAQ experiment and un-quantified in the other iTRAQ experiment.

Gene Ontology (GO) analysis was conducted using Blast2GO program against the non-redundant protein database. And the function characterization, biological network, pathway analysis were performed using Ingenuity Pathway Analysis software (IPA, www.ingenuity.com).

### Protein extraction and western blotting

Total protein was extracted using cell lysis buffer (50 mM Tris, 150 mM NaCl, 1 mM EDTA, 1% Triton X-100). Equal amounts of cell lysates were separated by 10% SDS-PAGE. Proteins were then transferred onto PVDF membranes (16916600, Roche), blocked with 5% non-fat milk in Tris-buffered saline containing 0.05% Tween 20 for 1 h, and probed with primary antibodies against ADH1C (A8081, ABclonal Technology), ALDH2 (A1226, ABclonal Technology), ALDH1A1 (A1802, ABclonal Technology), and β-Actin (4967L, Cell Signaling Technology). The specific proteins were detected with HRP-conjugated secondary antibodies (sc-2004, Santa Cruz Biotechnology), developed with SuperSignal West Pico Chemiluminescent Substrate (34080, Thermo Scientific) and visualized by Kodak Image Station 2000MM. Western blot results were quantified using Image J software.

### ADH and ALDH activity assay

The ADH activity was measured spectrophotometrically by monitoring the reductive reaction of NAD^+^ to NADH at 340 nm using Alcohol Dehydrogenase Assay Kit (A083, Nanjing Jiancheng Bioengineering Institute, China) in accordance with the manufacturer’s instructions. The ALDH activity was determined as previously^24^. Reaction rates were expressed as μmol NADH/min/mg protein. All data are representative of four independent experiments.

### MTT assay

The thiazolyl blue tetrazolium bromide (MTT) assay was performed using MTT Cell Proliferation and Cytotoxicity Assay Kit (E606334, Sangon Biotech, China) in accordance with the manufacturer’s instructions. Briefly, HepG2 cells were seeded at a density of 5 × 10^3^ cells/well on 96-well culture plates with ten replicates for each treatment, and incubated for 24 h at 37 °C under 5% CO_2_. Then the cells were treated with 100 mM ethanol to induce cytotoxicity in HepG2 cells^44^,^45^, coupled with 10 mM Arg treatment for 24 h. Cell morphology was observed by light microscopy. Then MTT reagent and formazan solubilization solution were added into the cells, followed by the measurement of the absorbance at 570 nm using a microplate reader.

### Statistical analysis

All statistical analyses were performed using GraphPad Prism software (version, 6.0c). Values are showed as mean ± standard deviation (SD). Differences were considered statistically significant at *P* < 0.05.

## Additional Information

**How to cite this article**: Yan, G. *et al*. Comparative Proteomics Analysis Reveals L-Arginine Activates Ethanol Degradation Pathways in HepG2 Cells. *Sci. Rep*. **6**, 23340; doi: 10.1038/srep23340 (2016).

## Supplementary Material

Supplementary Information

## Figures and Tables

**Table 1:** Amino acid metabolism-related differentially expressed proteins.

Protein name	Gene name	UniprotKB Acession	Ratio (+Arg/Ctrl)	AA metabolism process
Glycine N-methyltransferase	*GNMT*	Q14749	9.640	Glycine, serine and threonine metabolism
S-Adenosylmethionine synthase	*MAT1A*	A8K455	8.566	Cysteine and methionine metabolism
Regucalcin	*RGN*	Q15493	5.861	Valine, leucine and isoleucine metabolism
Dimethylglycine dehydrogenase	*DMGDH*	B3KQ84	5.155	Glycine, serine and threonine metabolism
Histidine ammonia-lyase	*HAL*	P42357	5.066	Histidine metabolism
3-Hydroxyisobutyrate dehydrogenase, mitochondrial	*HIBADH*	P31937	1.428	Valine, leucine and isoleucine degradation
