# Supplementary material for: Comparative Proteomics Analysis Reveals L-Arginine Activates Ethanol Degradation Pathways in HepG2 Cells
Source: Sci Rep. 2016 Mar 17;6:23340. doi: 10.1038/srep23340 (PMC4794764; doi:10.1038/srep23340)
Supplement: Supplementary Information [file srep23340-s1.pdf]

Supplementary Information:

Comparative proteomics analysis reveals L-arginine activates ethanol degradation pathways in HepG2 cells

Guokai Yan[1,2,*], Retno Lestari[3,*], Baisheng Long[1,2], Qiwen Fan[1,2], Zhichang Wang[1,2], Xiaozhen Guo[4], Jie Yu[1,2], Jun Hu[1,2], Xingya Yang[1,2], Changqing Chen[1,2], Lu Liu[1,2], Xiuzhi Li[1,2], Agung Purnomoadi[3], Joelal Achmadi[3] & Xianghua Yan[1,2]

[1]College of Animal Sciences and Technology, Huazhong Agricultural University, Wuhan, 430070, Hubei, China

[2]The Cooperative Innovation Center for Sustainable Pig Production, Wuhan, 430070, Hubei, China

[3]Faculty of Animal and Agricultural Sciences, Diponegoro University, Tembalang Campus, Semarang 50275, Central Java, Indonesia

[4]State Key Laboratory of Agricultural Microbiology, College of Veterinary Medicine, Huazhong Agricultural University, Wuhan, 430070, Hubei, China

[*]These authors contributed equally to this work

Correspondence and requests for materials should be addressed to X. Y. (email: xhyan@mail.hzau.edu.cn)

Content:

Supplementary Figure S1: Page 2

Supplementary Figure S2: Page 3

Supplementary Table S1: Page 4

Supplementary Table S2: Pages 5-7

Supplementary Figure S1:

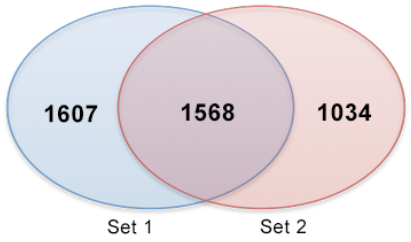

Supplementary Figure S1. Venn diagram of the number of quantified proteins derived from the two sets of iTRAQ experiments.

Supplementary Figure S2:

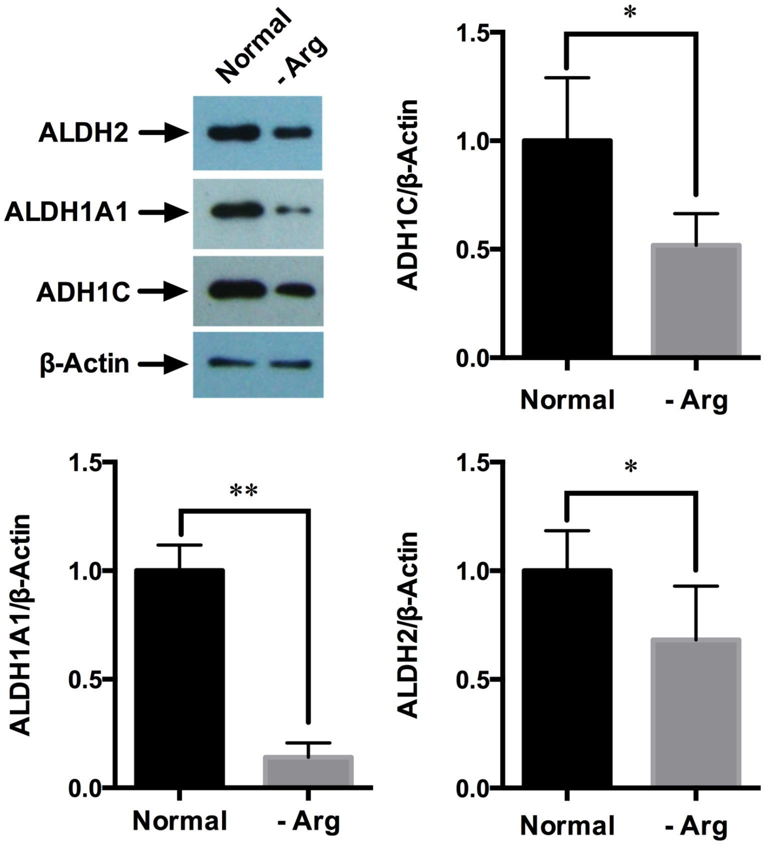

Supplementary Figure S2. Western blot of ADH1C, ALDH2, ALDH1A1, and β-Actin in HepG2 cells treated by normal and Arg deprived medium cultured HepG2 cells as indicated. Quantification of ADH1C/β-Actin, ALDH2/β-Actin, and ALDH1A1/β-Actin (normalized) was performed using Image J software. Data are means ± SD (n = 3). *$P < 0.05$, **$P < 0.01$ (Student's *t*-test).

Supplementary Table S1: Information of the 21 differentially expressed proteins identified by iTRAQ experiments.

Protein name	Gene symbol	Accession	Unique peptide (Set1|Set2)	% Sequence coverage (Set1|Set2)	Ratio used for analysis	Ratio (Set1|Set 2)
**HCG1745306, isoform CRA_a**	*HBA1/HBA2*	G3V1N2	3|-	19.1|-	82.766*	82.766|-
**Retinal dehydrogenase 1 (EC 1.2.1.36)**	*ALDH1A1*	B4DDF8	3|-	10.0|-	32.124*	32.124|-
**Alcohol dehydrogenase 1C**	*ADH1C*	P00326	3|-	6.4|-	14.530*	14.530|-
**Glycine N-methyltransferase**	*GNMT*	Q14749	3|-	12.9|-	9.640*	9.640|-
**Core histone macro-H2A.1**	*H2AFY*	O75367	3|-	12.6|-	9.305*	9.305|-
**S-adenosylmethionine synthase**	*MAT1A*	A8K455	3|-	21.3|-	8.566*	8.566|-
**Cytochrome P450, family 2, subfamily D, polypeptide 6**	*CYP2D6*	Q3KPF3	2|-	4.4|-	8.469*	8.469|-
**Delta-1-pyrroline-5-carboxylate dehydrogenase, mitochondrial**	*ALDH4A1*	P30038	3|-	6.2|-	7.710*	7.710|-
**Ribonuclease UK114 (Fragment)**	*HRSP12*	H0YB34	2|-	14.2|-	7.637*	7.637|-
**Aldehyde dehydrogenase family 8 member A1**	*ALDH8A1*	Q9H2A2	4|-	10.7|-	6.700*	6.700|-
**Long-chain-fatty-acid--CoA ligase 1 (EC 6.2.1.3)**	*ACSL1*	B4E0R0	2|-	2.6|-	6.186*	6.186|-
**Regucalcin**	*RGN*	Q15493	2|-	9.7|-	5.861*	5.861|-
**Mitochondrial aldehyde dehydrogenase 2 variant (Fragment)**	*ALDH2*	Q53FB6	11|-	30.8|-	5.305*	5.305|-
**Dimethylglycine dehydrogenase, mitochondrial (EC 1.5.99.2)**	*DMGDH*	B3KQ84	6|-	9.5|-	5.155*	5.155|-
**Histidine ammonia-lyase**	*HAL*	P42357	3|-	5.2|-	5.066*	5.066|-
**WD repeat-containing protein 81**	*WDR81*	Q562E7	3|1	4.6|1.7	1.773	2.304|1.242
**3-Hydroxyisobutyrate dehydrogenase, mitochondrial**	*HIBADH*	P31937	4|3	18.5|15.2	1.428	1.653|1.203
**ATP-dependent 6-phosphofructokinase, liver type**	*PFKL*	P17858	6|4	17.3|12.2	1.396	1.479|1.313
**Integrin alpha-3**	*ITGA3*	B4E0H8	3|12	3.5|13.2	1.341	1.369|1.312
**Cadherin-2**	*CDH2*	P19022	4|6	7.1|9.7	1.339	1.205|1.472
**Signal peptidase complex subunit 2**	*SPCS2*	A0A087WUC6	3|3	16.7|21.6	1.315	1.314|1.316

*Proteins quantified in one set of iTRAQ experiment while un-quantified in the other set of iTRAQ experiment, with ratio > 5 (< 0.2).

Supplementary Table S2: Functional characterization details of the differentially expressed proteins using the IPA tools.

Molecular and cellular function			
Category	*P*-value	Molecules	# Molecules
Lipid Metabolism	2.80E-08	ACSL1, ADH1C, ALDH1A1, ALDH8A1, CYP2D6, GNMT, H2AFY, ITGA3, RGN, MAT1A	10
Drug Metabolism	2.80E-08	ACSL1, ADH1C, ALDH1A1, ALDH2, ALDH8A1, CYP2D6, MAT1A	7
Small Molecule Biochemistry	2.80E-08	ACSL1, ADH1C, ALDH1A1, ALDH2, ALDH8A1, CYP2D6, DMGDH, GNMT, H2AFY, HAL, HBA1/HBA2, HIBADH, ITGA3, MAT1A, PFKL, RGN	16
Vitamin and Mineral Metabolism	2.80E-08	ADH1C, ALDH1A1, ALDH8A1, CYP2D6, RGN	5
Post-Translational Modification	5.49E-07	ALDH1A1, ALDH2, GNMT, MAT1A, PFKL	5
Protein Synthesis	5.49E-07	ALDH1A1, CDH2, GNMT, HBA1/HBA2, HRSP12, MAT1A, PFKL	7
Amino Acid Metabolism	9.69E-07	DMGDH, GNMT, HAL, HIBADH, MAT1A, RGN	6
Molecular Transport	2.96E-06	ACSL1, ADH1C, ALDH1A1, ALDH2, ALDH8A1, GNMT, H2AFY, HBA1/HBA2, ITGA3, MAT1A, RGN	11
Energy Production	5.92E-06	ACSL1, ADH1C, ALDH1A1, CYP2D6, HBA1/HBA2	5
Nucleic Acid Metabolism	9.85E-06	ALDH2, CYP2D6, GNMT, MAT1A	4
Cell Death and Survival	8.79E-04	ALDH1A1, ALDH2, CDH2, GNMT, HBA1/HBA2, ITGA3, RGN, WDR81	8
Cell Morphology	1.02E-03	CDH2, HBA1/HBA2, ITGA3, RGN	4
Cell-To-Cell Signaling and Interaction	1.02E-03	CDH2, CYP2D6, ITGA3	3
Cellular Assembly and Organization	1.02E-03	CDH2, HBA1/HBA2, ITGA3, MAT1A	4
Cellular Compromise	1.02E-03	ACSL1, ALDH2, CDH2, CYP2D6, WDR81	5
Gene Expression	1.02E-03	HRSP12	1
Carbohydrate Metabolism	1.14E-03	ACSL1, ALDH1A1, ALDH2, GNMT, PFKL, RGN	6
Cellular Movement	2.04E-03	ALDH2, CDH2, ITGA3, RGN	4
RNA Post-Transcriptional Modification	2.04E-03	CYP2D6	1
Cell Signaling	3.35E-03	CDH2, ITGA3	2
Cellular Function and Maintenance	9.13E-03	CDH2, HBA1/HBA2, ITGA3	3
Cellular Development	1.43E-02	ALDH1A1, CDH2, HBA1/HBA2, ITGA3, RGN	5
Cellular Growth and Proliferation	1.43E-02	ALDH1A1, CDH2, HBA1/HBA2, HRSP12, ITGA3, RGN	6
Immune Cell Trafficking	1.92E-02	ITGA3	1
Free Radical Scavenging	2.12E-02	ALDH2, CYP2D6, HBA1/HBA2	3
Protein Degradation	2.52E-02	ALDH1A1	1

Diseases and disorders			
Category	*P*-value	Molecules	# Molecules
Psychological Disorders	2.54E-04	ADH1C, ALDH1A1, ALDH2	3
Gastrointestinal Disease	3.00E-04	ACSL1, ADH1C, ALDH1A1, ALDH2, CDH2, GNMT, H2AFY, HBA1/HBA2, HRSP12, ITGA3, MAT1A, RGN	12
Metabolic Disease	3.00E-04	ACSL1, ALDH1A1, ALDH2, ALDH4A1, DMGDH, GNMT, H2AFY, HAL, HBA1/HBA2, HRSP12, MAT1A, RGN	12
Organismal Injury and Abnormalities	3.00E-04	ACSL1, ADH1C, ALDH1A1, ALDH2, ALDH4A1, ALDH8A1, CDH2, CYP2D6, DMGDH, GNMT, H2AFY, HAL, HBA1/HBA2, HIBADH, ITGA3, MAT1A, RGN, WDR81	18
Hepatic System Disease	8.29E-04	ACSL1, ADH1C, ALDH1A1, GNMT, H2AFY, HBA1/HBA2, MAT1A, RGN	8
Inflammatory Disease	8.29E-04	ALDH2, GNMT, HBA1/HBA2, HRSP12, ITGA3, MAT1A	6
Cancer	1.02E-03	ACSL1, ALDH1A1, ALDH2, ALDH4A1, ALDH8A1, CDH2, CYP2D6, GNMT, H2AFY, HBA1/HBA2, HIBADH, ITGA3, PFKL, RGN	14
Cardiovascular Disease	1.02E-03	ACSL1, ALDH2, CDH2, CYP2D6, GNMT, HBA1/HBA2	6
Connective Tissue Disorders	1.02E-03	ALDH1A1, HBA1/HBA2, ITGA3, RGN	4
Dermatological Diseases and Conditions	1.02E-03	H2AFY, HAL, HIBADH, ITGA3	4
Developmental Disorder	1.02E-03	ALDH1A1, ALDH4A1, HBA1/HBA2, ITGA3, MAT1A, WDR81	6
Hematological Disease	1.02E-03	ALDH1A1, ALDH2, ALDH4A1, CYP2D6, DMGDH, HBA1/HBA2	6
Hereditary Disorder	1.02E-03	ACSL1, ADH1C, ALDH2, ALDH4A1, DMGDH, GNMT, HAL, HBA1/HBA2, ITGA3, MAT1A, WDR81	11
Neurological Disease	1.02E-03	ADH1C, ALDH1A1, ALDH2, CDH2, CYP2D6, RGN, WDR81	7
Renal and Urological Disease	1.02E-03	ACSL1, ALDH1A1, CYP2D6, HBA1/HBA2, ITGA3	5
Respiratory Disease	1.02E-03	ALDH2, ITGA3	2
Infectious Diseases	1.52E-03	CYP2D6, HBA1/HBA2, ITGA3	3
Endocrine System Disorders	2.48E-03	ACSL1, ALDH1A1, ALDH2, ALDH4A1, CYP2D6, GNMT, HBA1/HBA2, HRSP12, ITGA3, PFKL, WDR81	11
Immunological Disease	2.48E-03	ACSL1, ALDH1A1, ALDH2, DMGDH, HBA1/HBA2, HRSP12	6
Skeletal and Muscular Disorders	3.05E-03	ACSL1, ALDH1A1, ALDH2, ITGA3, RGN	5
Reproductive System Disease	5.83E-03	ALDH1A1, CDH2, CYP2D6, H2AFY, HBA1/HBA2, HIBADH, ITGA3	7
Ophthalmic Disease	8.12E-03	ALDH1A1, WDR81	2
Nutritional Disease	1.92E-02	ADH1C, RGN	2

Physiological system development and function			
Category	*P*-value	Molecules	# Molecules
Organ Morphology	2.47E-04	ADH1C, ALDH1A1, CDH2, GNMT, HBA1/HBA2, ITGA3, MAT1A, RGN	8
Organ Development	2.47E-04	ALDH1A1, GNMT, HBA1/HBA2, ITGA3, MAT1A, RGN	6
Embryonic Development	2.47E-04	ALDH1A1, CDH2, ITGA3, RGN	4
Organismal Development	2.47E-04	ALDH1A1, CDH2, GNMT, H2AFY, ITGA3, MAT1A, RGN	7
Tissue Development	2.47E-04	ALDH1A1, CDH2, HBA1/HBA2, HRSP12, ITGA3, RGN	6
Visual System Development and Function	2.47E-04	ALDH1A1, ALDH8A1, ITGA3	3
Hepatic System Development and Function	3.00E-04	GNMT, HBA1/HBA2, MAT1A, RGN	4
Digestive System Development and Function	8.29E-04	GNMT, HBA1/HBA2, ITGA3, MAT1A, RGN	5
Inflammatory Response	8.29E-04	ALDH1A1, ALDH2, GNMT, HBA1/HBA2, HRSP12, MAT1A	6
Cardiovascular System Development and Function	1.02E-03	ADH1C, ALDH2, CDH2, CYP2D6, HBA1/HBA2, ITGA3, RGN	7
Connective Tissue Development and Function	1.02E-03	ALDH1A1, ALDH2, CDH2, RGN	4
Nervous System Development and Function	1.02E-03	ADH1C, ALDH1A1, CDH2, HBA1/HBA2, ITGA3	5
Tissue Morphology	1.02E-03	ACSL1, ALDH1A1, ALDH2, CDH2, GNMT, ITGA3, RGN, WDR81	8
Tumor Morphology	1.02E-03	CDH2, RGN	2
Skeletal and Muscular System Development and Function	2.04E-03	ALDH1A1, ALDH2, CDH2, ITGA3, RGN	5
Renal and Urological System Development and Function	4.07E-03	ITGA3	1
Respiratory System Development and Function	5.08E-03	ALDH1A1, ITGA3, RGN	3
Hematological System Development and Function	5.68E-03	ALDH2, CYP2D6, GNMT, HBA1/HBA2, ITGA3	5
Hair and Skin Development and Function	6.09E-03	ITGA3, RGN	2
Reproductive System Development and Function	6.09E-03	ITGA3,	1
Behavior	1.01E-02	ADH1C, HBA1/HBA2	2
Cell-mediated Immune Response	1.92E-02	ITGA3	1
Hematopoiesis	1.92E-02	ITGA3	1
